# Partner support in a cohort of African American families and its influence on pregnancy outcomes and prenatal health behaviors

**DOI:** 10.1186/1471-2393-13-187

**Published:** 2013-10-17

**Authors:** Jennifer K Straughen, Cleopatra H Caldwell, Alford A Young, Dawn P Misra

**Affiliations:** 1 Department of Family Medicine and Public Health Sciences, Division of Population Health Sciences, School of Medicine, Wayne State University, Detroit, MI, USA; 2 Program for Research on Black Americans, Institute for Social Research Department of Health Behavior and Health Education, School of Public Health, University of Michigan, Ann Arbor, MI, USA; 3 Department of Afroamerican and African Studies, University of Michigan, Ann Arbor, MI, USA; 4 Department of Sociology, University of Michigan, Ann Arbor, MI, USA

**Keywords:** Race/ethnicity, Paternal support, Preterm birth, Low birth weight, Maternal health behaviors

## Abstract

**Background:**

We examined how two indicators of partner involvement, relationship type and paternal support, influenced the risk of pregnancy outcomes (preterm birth, low birth weight) and health behaviors (prenatal care, drug use, and smoking) among African American women.

**Methods:**

Interview and medical record data were obtained from a study of 713 adult African American women delivering singletons between March 2001 and July 2004. Women were enrolled prenatally if they received care at one of three Johns Hopkins Medical Institution (JHMI) prenatal clinics or post-partum if they delivered at JHMI with late, no or intermittent prenatal care. Relationship type was classified as married, unmarried/cohabitating, or unmarried/non-cohabitating. Partner support was assessed using an 8-item scale and was dichotomized at the median. Differences in partner support by pregnancy outcome and health behaviors were assessed using linear regression. To assess measures of partner support as predictors of adverse pregnancy outcomes and health behaviors, Poisson regression was used to generate crude and adjusted prevalence ratios (PR) and 95% confidence intervals (CI).

**Results:**

There were no statistically significant differences in pregnancy outcomes or health behaviors by relationship type or when partner support was examined as a continuous or categorical variable. Modeled as a dichotomous variable, partner support was not associated with the risk of preterm birth (PR = 0.81, 95% CI = 0.56, 1.56), low birth weight (PR = 0.77, 96% CI = 0.48, 1.26), or health behaviors.

**Conclusions:**

Paternal involvement was not associated with pregnancy outcomes or maternal health behaviors. Attention to measurement issues and other factors relevant for African American women are discussed.

## Background

Racial disparities in adverse pregnancy outcomes continue to be a hallmark of births in the United States in spite of efforts to reduce these gaps. The infant mortality rate of Black infants (12.90/1000 live births) is more than twice that of white infants (5.57/1000 live births) [1]. A significant proportion of this disparity may be related to preterm birth. The preterm-related infant mortality rate for non-Hispanic Black women was 6.01/1000 live births whereas among non-Hispanic white women it was 1.79/1000 live births in 2006 [1]. Similarly, non-Hispanic Black mothers have a higher percentage of low birth weight infants (14%) than non-Hispanic whites, or Hispanics (11.7%, 12.2%, respectively). Low birth weight infants, preterm or not, have higher infant mortality rates than their normal weight counterparts, thus this also contributes to the disparities in infant mortality [1].

It is unclear why Black women are disproportionately affected by adverse pregnancy outcomes, but recent reports have highlighted our lack of attention to the role of the father in pregnancy [2,3]. The contribution and role that Black fathers may play in families may be different from that of white fathers as many Black fathers face unique, yet highly interconnected, barriers to involvement including joblessness, low educational attainment, and declining marriage rates [3]. Most studies to date have used surrogate indicators of paternal involvement, such as marital status and presence of father’s name on the birth certificate [2,4-6]. A few studies have considered the nature of the mother-father relationship (e.g. unmarried, but cohabiting or unmarried and non-cohabitating) for pregnancy outcomes with mixed results. For example, unmarried women have a higher proportion of low birth weight infants then their married counterparts in studies not considering race or ethnicity as a contextual factor that might influence this association [7,8]. On the other hand Thorburn Bird et al., focusing on racial and ethnic subgroups within their sample, found that non-Hispanic Black women had similar risks of delivering a low birth weight infant, regardless of marital relationship type (married, cohabitating, or other) [9]. A smaller number of studies have examined other aspects of father involvement, such as father’s attitude and behaviors regarding the pregnancy and the mother’s perception of the father’s support [8,10,11]. The findings of these studies were mixed with some measures, such as greater partner happiness with the pregnancy, being associated with decreased birth weight, but other measures, such as paternal intendedness, were not associated with birth weight [11]. The Fragile Families study, a large study of vulnerable families that examined multiple measures of paternal involvement, reported that women who received financial support from fathers prenatally had higher rates of prenatal care [10]. Associations between maternal health behaviors and relationship type have also been noted from this study [10,12]. For example, women who reported good relationships with the father of their baby were less likely to smoke or use drugs than those reporting fair or poor quality of relationships [12]. It is unknown the extent to which these findings are truly representative of father’s involvement, depending on relatively blunt and sometimes indirect (reported from only the mother) measures of partner support and relationships. While these proxy indicators are suggestive, the specific aspects of father’s involvement that confer benefit remain unclear.

The current state of research on racial and ethnic disparities in birth outcomes highlights the need to identify factors that may be more salient to experiences within Black families in effort to reduce these disparities. The involvement of fathers in the pregnancy experience may be a promising direction because of previous research suggesting an indirect influence of fathers on mothers’ health behaviors. How Black fathers may influence pregnancy outcomes and maternal health behaviors is unclear, especially because of the interconnections and support behaviors between members of extended family systems often found for Black families. Further, many Black males in the United States have a disadvantaged economic position, a key factor in declining marriage rates [13]; consequently, marital status may be reflective of their socioeconomic position, rather than the quality of the mother-father relationship. Thus, within Black families, marital status may not be a reliable indicator of father involvement during pregnancy. Moreover, there has been a failure to consider the possibility that volatile relationships could negatively impact pregnancy outcomes. In summary, better characterization of the quality of the mother-father relationship during pregnancy in Black families is needed as a way to better understand birth outcomes. We undertook these analyses in order to further characterize how two measures of partner involvement often used in birth outcomes research, relationship type based on marital status and cohabitation and partner support reported by the mother, influenced the risk of preterm birth, low birth weight, and maternal health behaviors in a low income cohort of Black women. We hypothesized that women with more involved partners would have better pregnancy outcomes and more favorable health behaviors than women with less involved partners. An additional goal was to assess the potential usefulness of broad partner involvement measures in understanding the influence of complex social relations during pregnancy among Black women.

## Methods

Study participants were enrolled as part of a study of preterm birth among African American women between March 2001 and July 2004. The study was reviewed and approved by institutional review boards of both The Johns Hopkins University School of Public Health and the University of Michigan School of Public Health. Women were eligible for participation if they received prenatal care at one of three Johns Hopkins Medical Institution’s clinics or if they delivered at Johns Hopkins Hospital after receiving late or no prenatal care. Women were enrolled prenatally between 22 and 28 weeks of gestation or postnatally. Informed consent was obtained from all study participants. The response rate was 68%. Women who were enrolled prenatally were interviewed in person twice, once prenatally and once postnatally. For women enrolled prenatally, data on partner support, race/ethnicity, age, education, locus of control, social support, marital status, anxiety, stress, unemployment, smoking, alcohol, and drug use were collected as part of the prenatal interview. The shorter postpartum interview asked women about sexual history, services received during pregnancy, and abuse. Women who were enrolled postnatally were interviewed once (during the postpartum hospitalization) to measure factors from the duration of the pregnancy. Therefore, this interview sought to capture all the data that was captured by the two interviews administered to the women enrolled prenatally. The interview for women enrolled during the postpartum period included data on partner support, race/ethnicity, age, education, locus of control, social support, marital status, anxiety, stress, unemployment, smoking, alcohol, drug use, sexual history, services received during pregnancy, and abuse. Data on medical history, pregnancy outcome, and pregnancy complications were ascertained from medical records.

In this analysis, only women who were 18 years of age or older who gave birth to a live-born singleton infant were eligible (n = 722 of the 842 participants). Outcomes of interest were: preterm birth, low birth weight, adequacy of prenatal care, prenatal smoking, and prenatal drug use. Preterm birth was defined as birth at less than 37 weeks of gestation and low birth weight encompassed all infants that were less than 2500 grams at birth. Gestational age based on last menstrual period as recorded in the medical record was systematically compared with other estimates of gestational age in a hierarchical fashion [14]. When the gestational age estimates were inconsistent, the initial estimate by the provider based on early ultrasound was used.

Prenatal smoking and drug use were based on maternal self report and classified as yes or no. Women were considered smokers if they reported smoking in the second trimester. Adequacy of prenatal care was determined by medical record abstraction and computation of the Adequacy of Prenatal Care Utilization Index [15]. We considered the five predefined classifications (adequate plus, adequate, intermediate, inadequate, and unknown), but ultimately collapsed the intermediate, inadequate, and unknown prenatal care groups as we were interested in receipt of at least adequate prenatal care. Those with unknown prenatal care were included in this group because our previous research in this population indicated that this group was a high risk group with little or no access to prenatal care.

Marital status and cohabitation were based on maternal self-report. Marital status and cohabitation were used to construct three categories for the relationship type variable: married unmarried and cohabitating, and unmarried and non-cohabitating. Partner support was assessed using an 8-item scale (Cronbach’s α = 0.95) that inquired about emotional and financial support as well as other factors such as dependability and assistance with childcare. For women enrolled prenatally, the scale was asked generally, not with regard to a specific period in pregnancy. For women enrolled postpartum, the scale was necessarily used at a later time point but again was not asked with regard to a specific period in pregnancy. The instructions and a list of the items included in the partner support scale is provided in Table 1. Response choices for each question included: strongly agree, agree, disagree, strongly disagree, or don’t know. The theoretical range for this scale is 8–32 (sample range 8–32). Item specific missing values for the 8-item partner’s support scale were imputed using the mean of the non-missing scale items for each study participant. Women who were missing all 8 responses for the scale were excluded from that portion of the analysis (n = 64). A majority of these 64 women (95.3%) did not have a regular partner or fiancé. The partner support questions were not asked of the women who reported not having a regular partner or fiancé. Partner’s support was classified into supportive and unsupportive based on the median scale score.

**Table 1:** Items included in the 8-item partner support scale

	**Please tell me whether you strongly agree, agree, disagree, or strongly disagree with the following statements about your partner**				
		**Strongly agree**	**Agree**	**Disagree**	**Strongly disagree**
1.	My partner is someone I can count on for financial support if I need it	4	3	2	1
2.	My partner is someone I can talk with about things that are important to me	4	3	2	1
3.	My partner is someone who is affectionate toward me	4	3	2	1
4.	My partner is someone who helps me care for my child(ren)	4	3	2	1
5.	My partner is someone who understands how I am feeling	4	3	2	1
6.	My partner is someone who talks with me and spends time with me	4	3	2	1
7.	My partner is someone whom I can count on	4	3	2	1
8.	My partner is someone who does things with me	4	3	2	1

Covariates were classified as follows: education (≥ high school education or equivalent or < high school education), Medicaid (yes or no), prenatal physical abuse (yes or no), emotional abuse (yes/no), control/dominance (yes or no), and welfare services (yes or no). Maternal age, the 12- item daily hassles scale, the Center for Epidemiologic Studies Depression scale (CES-D), and the Family Resource Scale were treated as continuous variables [16,17]. The Family Resource Scale is a 25 item instrument assessing adequacy of resources across several dimensions, including time and money needed for necessities (e.g., rent, heating) as well as for non-necessities (e.g., toys, vacation, restaurant meals) [17,18]. For each item, study participants indicated how often they had enough resources (on a five-point scale from ‘almost always to almost never’) during the past year with high scores reflecting inadequate resources (theoretical range 25–125).

Demographic and social variables as well as measures of partner support were compared using Chi square tests and analysis of variance as appropriate to test for differences by relationship type. Linear regression was used to evaluate differences in partner’s support (as a continuous outcome) and each of the pregnancy outcomes and maternal health behavior indicators (preterm birth, low birth weight, prenatal care, drug use, and prenatal smoking).

In order to examine relationship type and perceived partner support as predictors of adverse pregnancy outcomes and maternal prenatal health behaviors, Poisson regression with robust error variance was used to generate crude and adjusted prevalence ratios (PR) and their associated 95% confidence intervals (CI) [19]. Some of our outcomes do not meet the rare disease assumption of logistic regression; therefore prevalence ratios as opposed to odds ratios were calculated. Covariates were evaluated as potential confounders if previous studies suggested they were important or if they were associated with the outcome of interest or relationship type. Potential confounders were retained in the model if the coefficient for the exposure of interest changed by more than 5% upon addition of the covariate to the model either alone or in combination with other potential confounders. Due to the very small number of divorced and separated women (n = 9), these women were excluded from analyses. As a result, the final sample included 713 women. All statistical tests were two-tailed with a type 1 error rate fixed at 5 percent. SAS version 9.1 (SAS Institute, Cary, NC) was used to perform all analyses.

## Results

Approximately half of the 713 women included in this is study were enrolled prenatally (54.7%) with the remaining being enrolled postnatally (45.3%). A majority of the women were unmarried and not cohabiting with their partner (Table 2). Married women constituted 10.9% of the study population, 37.7% were unmarried and cohabitating, and 51.3% were unmarried and non-cohabitating. Timing of enrollment did not differ by relationship type (p = 0.81). Married women tended to be older and more educated than their unmarried counterparts. Although married women were less likely to use welfare than unmarried women, the majority of the study population was of low socioeconomic status. About 23% of married women and almost 50% of unmarried women regardless of cohabitation status utilized welfare services during pregnancy. Similar proportions of married and unmarried women received Medicaid assistance. Overall, more than 12% of the women had a low birth weight infant and 16.7% had preterm delivery.

**Table 2:** Distribution of maternal and infant characteristics by relationship type

	**Married**		**Unmarried, cohabitating**		**Unmarried, non**-**cohabitating**		**p-value**
	**N = 78**		**N = 269**		**N = 366**		
	**Mean**	**+/−**	**Mean**	**+/−**	**Mean**	**+/−**	
Maternal age*	28.12	5.12	23.62	5.15	23.51	5.15	<.0001
Family Resources Scale*	44.34	15.17	45.07	13.40	49.29	15.99	0.0006
	**n**	**%**	**n**	**%**	**n**	**%**	**p-value**
High school education or equivalent*							0.01
Yes	56	71.79	158	58.74	192	52.46	
No	22	28.21	111	41.26	174	47.54	
Smoking status^a^							0.55
Yes	11	14.10	56	20.82	73	19.95	
No	67	85.90	211	78.44	292	79.78	
Unknown	0	0	2	0.74	1	0.27	
Drug use*							0.02
Yes	7	8.97	28	10.41	63	17.21	
No	71	91.03	241	89.59	303	82.79	
Welfare services*							0.0004
Yes	18	23.08	134	49.81	178	48.63	
No	56	71.79	122	45.35	176	48.09	
Unknown	4	5.13	13	4.83	12	3.28	
Medicaid							0.12
Yes	40	51.28	164	60.97	244	66.67	
No	34	43.59	93	34.57	110	30.05	
Unknown	4	5.13	12	4.46	12	3.28	
Physical abuse							0.48
Yes	0	0	7	2.60	11	3.01	
No	74	94.87	248	92.19	342	93.44	
Unknown	4	5.13	14	5.20	13	3.55	
Emotional abuse							0.24
Yes	8	10.26	44	16.36	59	16.12	
No	65	83.33	192	71.38	259	70.77	
Unknown	5	6.41	33	12.27	48	13.11	
Control/dominance							0.15
Yes	9	11.54	54	20.07	63	17.21	
No	64	82.05	182	67.66	255	69.67	
Unknown	5	6.41	33	12.22	48	13.11	
Adequacy of prenatal care							0.71
Adequate or adequate plus	29	37.18	82	30.48	98	26.78	
Intermediate	7	8.97	28	10.41	38	10.38	
Inadequate	25	32.05	92	34.20	136	37.16	
Unknown	17	21.79	67	24.91	94	25.68	
Preterm							0.72
Yes	14	17.95	41	15.24	64	17.49	
No	64	82.05	228	84.76	302	82.51	
Low birth weight							0.59
Yes	7	8.97	36	13.38	44	12.02	
No	69	88.46	230	85.50	319	87.16	
Unknown	2	2.56	3	1.12	3	0.82	

^a^based on self-report of smoking in the 2nd trimester.

*Statistically significant differences between relationship types.

### Relationship type

#### Marital status/cohabitation

When compared to married women, unmarried women, regardless of cohabitation status, did not have an increased risk of preterm delivery (adjusted PR for cohabitating and unmarried non-cohabitating women respectively: PR = 0.80, 95% CI = 0.44, 1.48 and PR = 0.95, 95% CI = 0.53, 1.69) or low birth weight (adjusted PR for cohabitating and unmarried, non-cohabitating women respectively: PR = 1.91, 95% CI = 0.77, 4.73 and PR = 1.79, 95% CI = 0.72, 4.48) in adjusted or unadjusted analyses (Table 3). Relationship type was not associated with receipt of adequate prenatal care (PR for cohabitating and unmarried, non-cohabitating women respectively: PR = 0.82, 95% CI = 0.58, 1.15 and PR = 0.72, 95% CI = 0.52, 1.01). Similarly, relationship type was not associated with prenatal drug use (adjusted PR for cohabitating and unmarried, non-cohabitating women respectively: PR = 0.78, 95% CI = 0.34, 1.79 and PR = 1.17, 95% CI = 0.53, 2.59). Abuse did not appear to account for the lack of association as the different types of abuse were evaluated as possible confounders. Abuse was a confounder is the association with low birth weight, but its inclusion did not markedly alter the point estimate. While crude analyses were suggestive, there were no statistically significant differences in prenatal smoking between married and cohabitating women (PR = 1.81, 95% CI = 0.99, 3.34) or unmarried, non-cohabitating women (PR = 1.27, 95% CI = 0.71, 2.30) in adjusted analyses.

**Table 3:** **The association between relationship type**, **pregnancy outcomes**, **and maternal health behaviors**

	**Married**		**Cohabitating**				**Unmarried, Non**-**cohabitating**			
	**N = 78**		**N = 269**				**N = 366**			
			**Crude**		**Adjusted**		**Crude**		**Adjusted**	
**Pregnancy outcome**/**health behavior**	**PR**^**a**^	**95% CI**^**b**^	**PR**	**95% CI**	**PR**	**95% CI**	**PR**	**95% CI**	**PR**	**95% CI**
Preterm^c^	Referent	--	0.85	0.49, 1.47	0.80	0.44, 1.48	0.97	0.58, 1.65	0.95	0.53, 1.69
Low birth weight^d^	Referent	--	1.47	0.68, 3.17	1.91	0.77, 4.73	1.32	0.62, 2.81	1.79	0.72, 4.48
Adequate prenatal care^e^	Referent	--	0.82	0.58, 1.15	0.82	0.58, 1.15	0.72	0.52, 1.01	0.72	0.52, 1.01
Prenatal smoking^f^	Referent	--	1.49	0.82, 2.70	1.81	0.99, 3.34	1.42	0.79, 2.55	1.27	0.71, 2.30
Prenatal drug use^g^	Referent	--	1.16	0.53, 2.55	0.78	0.34, 1.79	1.92	0.91, 4.03	1.17	0.53, 2.59

^a^PR = prevalence ratio.

^b^CI = confidence interval.

^c^Adjusted prevalence ratio controls for maternal age, Medicaid, welfare, and smoking.

^d^Adjusted prevalence ratio controls for the Family Resource Scale, Medicaid, welfare, smoking, and physical abuse, emotional abuse, and control/dominance.

^e^Adjusted estimates are the same as the unadjusted as there were no significant confounders.

^f^adjusted estimates control for maternal age, maternal education, drug use, welfare, and the family resource scale.

^g^Adjusted estimates control for maternal age, maternal education, the family resource scale, Medicaid, welfare, and smoking.

#### Partner support

**Figure 1:**
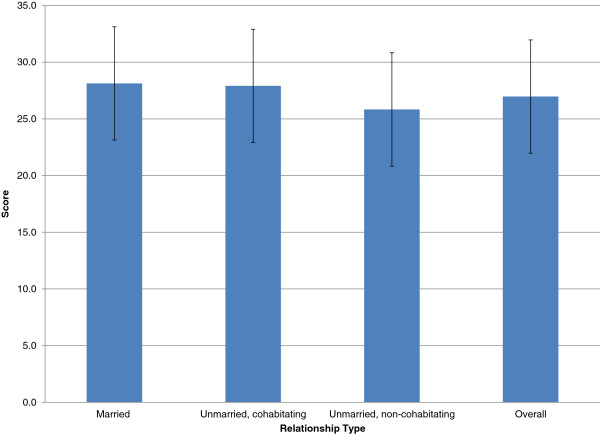
**Mean partner support scale score overall and by relationship type (p < 0**.**0001).**

Mean partner support was evaluated as a continuous outcome using linear regression. Mean partner support score by relationship type is presented in Figure 1. There were no differences in scale score total by preterm birth (p = 0.37), low birth weight (p = 0.56), prenatal care use (p = 0.46), drug use (p = 0.10), or prenatal smoking (p = 0.19). We also compared birth outcomes and health behaviors using a dichotomous variable defined as support greater or equal to or less than the median support score (Table 4). No associations between preterm birth and perceived partner support were found even after controlling for potential confounders. Women who perceived their partners as supportive overall did not have a decreased risk of preterm birth (PR = 0.80, 95% CI = 0.56, 1.56). Similar findings were reported for the risk of low birth weight (PR = 0.77, 96% CI = 0.48, 1.26). When maternal health behaviors were examined in relation to perceived partner support, no significant associations were found. Women who reported more support from partners were no more likely to obtain adequate prenatal care than their counterparts with unsupportive partners (PR = 1.20, 95% CI = 0.94, 1.52). Similar results were obtained for prenatal smoking (PR = 0.91, 95% CI = 0.66, 1.26) and drug use (PR = 0.92, 95% CI = 0.61, 1.38). Timing of enrollment in the study (prenatal or postnatal) was examined as a potential confounder. Timing of enrollment differed by partner support status, as might have been expected given that women enrolling postpartum had late or no care by design, but controlling for timing of enrollment did not impact our effect estimates.

**Table 4:** The association between selected pregnancy outcomes and health behaviors for supportive versus unsupportive partners (referent)

	**Unsupportive partners**^**a **^**N = 318**		**Supportive partners N = 331**			
			**Crude**		**Adjusted**	
**Pregnancy outcome**/**health behavior**	**PR**^**b**^	**95% CI**^**c**^	**PR**	**95%CI**	**PR**	**95%CI**
Preterm^d^	Referent	--	0.74	0.52, 1.05	0.80	0.56, 1.15
Low birth weight^e^	Referent	--	0.71	0.47, 1.08	0.77	0.48, 1.26
Adequate prenatal care^f^	Referent	--	1.13	0.89, 1.44	1.20	0.94, 1.52
Prenatal smoking^g^	Referent	--	0.81	0.59, 1.12	0.91	0.66, 1.26
Prenatal drug use^h^	Referent	--	0.73	0.48, 1.10	0.92	0.61, 1.38

^a^Using the median score on the partner support scale as the cut-point.

^b^PR = prevalence ratio.

^c^CI = confidence interval.

^d^Adjusted for the family resource scale.

^e^Adjusted for emotional abuse and the family resource scale.

^f^Adjusted for Medicaid and welfare.

^g^Adjusted for Medicaid, welfare, the family resource scale, and drug use.

^h^Adjusted for the family resource scale and smoking.

A subset of women reported that their current partner was not the father of the baby for her current pregnancy (n = 84); therefore we excluded these women and re-ran all analyses. There were no significant differences in the risk of preterm birth or low birth weight or in any of the health behaviors with regard to relationship type or perceived partner support (data not shown).

## Discussion

Our large study of low income Black women did not find any associations between relationship type or perceived global partner support with preterm birth, low birth weight, or maternal health behaviors. These findings agree with some, but not all previous studies, although many used different indicators of partner or father involvement than used here. Furthermore, within studies, findings may vary depending on how partner or father involvement is defined. Differences in defining and measuring partner or father involvement may explain discrepant results. In a birth certificate records based study in Milwaukee, Wisconsin, investigators reported an increased risk of preterm delivery among unmarried African American women who did not report the father on the birth certificate when compared to married African American women (OR = 1.41, 95% CI = 1.30, 1.35) [20]. However, in this same study, there was no difference in preterm delivery risk between married and unmarried African American women in cases where there was a paternity statement or court established paternity for the unmarried couples [20]. Similar patterns were found for low birth weight risk. For example, one report used Florida vital statistics records to examine paternal involvement based on presence or absence of father’s information on the birth certificate. The authors found that Black mothers with absent fathers as well as Black mothers with present fathers were more likely to deliver preterm and were more likely to have a low birth weight infant than white mothers with a present father [2]. That study used surrogate measures of father’s presence that were based on presence or absence of the father’s first and/or last name on the birth certificate [2].

In our analyses, partner involvement as measured by relationship type or the partner support scale was not associated with maternal health behaviors. This conflicts with the findings from the Fragile Families and Child Well-being study. That study found higher rates of prenatal care among women with involved fathers and lower rates of smoking and drug use among married as opposed to cohabitating or romantic, non-cohabitating relationships [10]. A more recent study focusing on the unmarried women within the Fragile Families cohort reported that women in poor relationships were more likely to report prenatal smoking and drug use than those in good relationships [12]. However, the results reported from Fragile Families did not stratify on race/ethnicity and may not have captured the unique experience of Black women. Differences among studies in terms of socioeconomic status, stress, and other psychosocial factors that may influence pregnancy outcome may also account for the disparate findings.

The use of surrogate measures of involvement and the failure to consider the potential negative implications of fathers may also account for the results of our study. Like most studies, we used surrogate measures of paternal involvement, including an indirect relationship type measure as well as a more direct measure of paternal support. However, neither was associated with pregnancy outcomes or maternal health behaviors. Use of a dichotomous global partner support measure was less informative than perhaps examining the four functional types of support (e.g., emotional, instrumental, appraisal, informational) or a more extensive social networks strategy that considers negative influences of social relations on health [21,22]. An approach that utilized a more sensitive measure of partner support could have yielded different findings. We suggest that future studies use more comprehensive father involvement measures.

An important strength of our study was our ability to better classify relationship type based on self-reported cohabitation status and marital status and to do so within a cohort of Black women. This information refines our assessment of relationship type, particularly important given the very small proportion of married women in our cohort, paralleling lower rates of married Black mothers compared to white mothers [7]. Cohabitation may offer opportunities for involvement or support similar to marriage in this context.

In spite of our study’s strengths, we are limited by failing to collect any information directly from the partners. In this regard, our study is comparable to most published work in this area. The Fragile Families study is one of few studies that sought to collect data directly from fathers and even that study’s published results are based on the maternal reports. Be that as it may, it is currently unknown from our work and others whether it is perceived maternal support from partners or actual support that matters. Additional studies are needed to address this limitation. We were also unable to directly assess partner conflict except in the extreme case of violence (emotional and physical). Partner support and conflict are not typically considered opposite ends of the continuum. Rather, you can have both support and conflict in a relationship. It is when the loss of equilibrium occurs that the danger of stress may increase, with potential negative implications for birth outcomes. Low support may not trigger the same emotional response in the same physiological way as emotional conflict in a relationship [23]. Further, partner support questions were asked with regard to the entire prenatal period and were not assessed longitudinally. It is unknown how these factors may have changed toward the end of pregnancy and whether changes in partner support over the duration of the pregnancy impacted the risk of preterm birth or low birth weight.

## Conclusions

It remains unclear whether partner involvement can have a positive impact on pregnancy outcomes and maternal health behaviors as the studies to date have presented conflicting evidence. Nonetheless, racial and ethnic disparities in birth outcomes emphasize the need to examine whether maternal health behaviors and pregnancy outcomes can be improved with increased partner support and involvement, particularly among Black families. Our research team is currently collecting more nuanced and comprehensive data in a new study of preterm birth in Black women that we hope will begin to address these issues. Future studies are needed to determine whether interventions targeted at increasing paternal support can improve pregnancy outcome and if true, they need to be designed so that one can delineate the specific aspects of partner support that have a positive effect on pregnancy outcome.

## Abbreviations

CESD: Center for epidemiologic studies depression scale; PR: Prevalence ratios; CI: Confidence intervals.

## Competing interests

The authors declare that they have no competing interests.

## Authors’ contributions

JKS contributed to the study’s design, analyzed the data, and participated in the writing of the manuscript. CHC contributed the conception and design of the study and contributed to the writing and revision of the manuscript. AAY contributed the conception and design of the study and contributed intellectual content to the body of the manuscript. DPM contributed the conception and design of the study, assisted with the interpretation of the results, and contributed to the writing and revision of the manuscript. All authors read and approved the final manuscript.

## Pre-publication history

The pre-publication history for this paper can be accessed here:

http://www.biomedcentral.com/1471-2393/13/187/prepub
